# Abdominal Waist Circumference and Subcutaneous Adipose Tissue Thickness Predicts Development of Post-Transplant Diabetes Mellitus

**DOI:** 10.21203/rs.3.rs-8543193/v1

**Published:** 2026-04-05

**Authors:** Mehmet Kanbay, Feyyaz Yagmur, Bilge Karadeniz, Hande Ozen Atalay, Ahmet Ak, Sidar Copur, Helin Orak, Nur Genc, Nuri Hasbal, Gizem Kula, Afak Durur Karakaya, Peter Rossing, Adrian Covic, Katherine Tuttle

**Affiliations:** Koç University; Koç University; Koç University; Koç University; Koç University; Koç University; Koç University; Koç University; Koç University; Koç University; Steno Diabetes Center; Grigore T. Popa University of Medicine and Pharmacy; University of Washington

**Keywords:** Post-transplant diabetes mellitus, Subcutaneous adipose tissue, Waist circumference, Kidney transplantation

## Abstract

**Background:**

Post-transplant diabetes mellitus (PTDM) is a major comorbidity affecting 10–40% of kidney transplant recipients with significant clinical consequences including diabetic microvascular and macrovascular complications, infectious complications, allograft loss, and mortality. Although multiple modifiable and non-modifiable risk factors have been identified for PTDM development, there is a strong need for higher quality determinants of PTDM risk for early identification of high-risk patients. We hereby aim to evaluate the efficacy of abdominal waist circumference and subcutaneous adipose tissue thickness as predictors of PTDM among kidney transplant recipients.

**Methods:**

We have performed a single-centered retrospective clinical study involving non-diabetic kidney transplant recipients between December 2018 and January 2025. Baseline demographic and clinical data, laboratory workup and pre-transplant abdominal computed tomography (CT) had been utilized. Abdominal waist circumference and subcutaneous adipose tissue thickness have been obtained from abdominal CT scan. The diagnosis of PTDM was based upon the criteria established by the American Diabetes Association.

**Results:**

We have included a total of 478 adult kidney transplant recipients with a mean age of 41.1 with slight female predominance (57.1%). Patients developing PTDM were more likely to be at elderly age, have higher body-mass index, higher abdominal subcutaneous adipose tissue thickness, higher abdominal waist circumference and higher baseline serum glucose and triglyceride levels compared to patients not developing PTDM (p-value < 0.001 for all). The pairwise comparison of the ROC curve data for such variables has revealed the superiority of higher abdominal subcutaneous adipose tissue thickness and abdominal waist circumference in predicting PTDM risk over body-mass index among kidney transplant recipients.

**Conclusions:**

We have identified two independent risk factors novel for PTDM development as abdominal waist circumference and abdominal subcutaneous adipose tissue thickness.

## Introduction

Kidney transplantation is the gold standard therapeutic approach for the management of end-stage kidney disease (ESKD) [1]. Diabetes mellitus is the most common underlying etiology of end-stage kidney disease. For those without diabetes at the time of transplantation, a major complication is post-transplant diabetes mellitus (PTDM), defined as newly diagnosed diabetes after transplantation. However, diagnosis of PTDM within the first 45 days post-transplant is discouraged due to transient factors such as infections, induction immunosuppression, or acute rejection treatments [2, 3]. Currently, PTDM affects 10–20% of kidney transplant recipients with a strong association with premature major cardiovascular events, allograft loss and mortality [4, 5]. Multiple transplant-related risk factors including older age, obesity and metabolic syndrome, hepatitis C (HCV) or cytomegalovirus (CMV) infections, calcineurin inhibitor or mammalian target of rapamycin inhibitor therapy, corticosteroid therapy, acute rejection episodes, higher pre-transplant hepatic or pancreatic steatosis status have been identified acting through either pancreatic beta-cell dysfunction or peripheral insulin resistance [6, 7]. Even though obesity and high body-mass index (BMI) have been identified as risk factors for PTDM [8], BMI lacks specificity as it may not differentiate between different body compartments such as skeletal muscle and adipose tissue, distribution of adipose tissue or hypervolemia. As PTDM is a prevalent complication with devastating clinical consequences in the solid organ transplant population, the screening and pre-transplant risk stratification and/or potential preventive measures are at most importance [9]. We hereby aim to evaluate the efficacy of pre-transplant anthropometric measures including abdominal waist circumference and abdominal subcutaneous adipose tissue thickness in predicting PTDM risk among non-diabetic kidney transplant recipients.

## Materials & Methods

### Study Design:

We conducted a single-center retrospective cohort study involving kidney transplant recipients at the Koç University Hospital Solid Organ Transplant Center between December 2018 and January 2025. The inclusion criteria are as follows: (a) Being a kidney transplant recipient at or over age 18; (b) Availability of abdominal computed tomography (CT) scan within six months prior to transplantation; (c) Presence of post-transplant follow-up data for at least six months. Exclusion criteria include diagnosis of diabetes mellitus prior to kidney transplantation. All patients included in this retrospective study received rabbit anti-thymocyte globulin as part of an induction immunosuppression regimen with dose titration relative to CD3 cell count and a triple maintenance immunosuppressive regimen including calcineurin inhibitor (tacrolimus), anti-metabolite agent (mycophenolate mofetil) and low-dose prednisolone. Maintenance immunosuppressive regimens may be switched to include the mammalian target of rapamycin inhibitors (everolimus) or other anti-metabolite agents (azathioprine) in accordance with the adverse effects, individual patient risk assessment for rejection or other considerations including pregnancy or malignancies. Ethical approval for this study was obtained from the institutional board of ethics (Ethics Board Approval 2023.137.IRB1.048).

The diagnosis of PTDM was based upon the criteria established by the American Diabetes Association: (a) oral Glucose Tolerance Test: 2-h plasma glucose levels ≥ 200 mg/dL; (b) fasting plasma glucose ≥ 126 mg/dL; (c) random plasma glucose levels ≥ 200 mg/dL with the presence of diabetes symptoms; (d) hemoglobin A1c level > 6.5% measured methods with NGSP certified and standardized to the DCCT assay [10]. We utilized random plasma glucose with diabetes symptoms, hemoglobin A1C measurements and fasting plasma glucose levels in combinations of two to conclude on the diagnosis of PTDM, we did not use OGTT to diagnose any patients for PTDM. Transplant recipients’ blood samples are obtained 8.00–9.00AM in the morning with at least 8 hours of overnight fasting. Kidney transplant recipients meeting the mentioned diagnostic criteria and/or patients taking any anti-hyperglycemic medications at least after post-transplant 120 days were categorized as having PTDM.

### Metabolic Parameters:

The baseline medical history and laboratory workup of all kidney transplant recipients have been extensively evaluated. The medical records included in our analysis include chronic kidney disease etiology, comorbidities including hypertension and heart failure, smoking history and acute rejection episodes. The laboratory tests included in our analysis include complete blood count, kidney function tests including serum creatinine and estimated glomerular filtration rate (eGFR), serum lipid profile, liver function tests, plasma glucose, thyroid function tests, pre-transplant (a day before surgery) magnesium, post-transplant (a week later) magnesium, serological workup of donor and recipients anti-hepatitis B core antibody, anti-hepatitis C antibody, hepatitis B surface antigen, anti-cytomegalovirus (CMV) antibodies of donor and recipients. Estimated glomerular filtration rate (eGFR) had been calculated via the Chronic Kidney Disease Epidemiology Collaboration (CKD-EPI) formula considering age, race, gender, and serum creatinine level [11].

### Radiological Evaluation:

Pre-transplant evaluation was performed via abdominal CT with intravenous iodinated contrast material. Abdominal CT scans were conducted using a 256-slice multidetector scanner (Somatom Definition AS and Flash; Siemens Healthcare, Germany) with parameters of 120 kVp, 1.5-mm slice thickness, 1.5-mm reconstruction intervals, and a 512 × 512 matrix.

Two experienced radiologists analyzed non-contrast abdominal CT images on consensus. They were unaware of the patients’ clinical information during the assessment.

Abdominal circumference and subcutaneous adipose tissue thickness were calculated. The abdominal circumference was accepted as the outer contour length of the abdominal skin and calculated on the axial slice located at the midpoint between the inferior margin of the lowest rib and the superior margin of the iliac crest [12]. Abdominal subcutaneous fat thickness was assessed at the umbilical level [13]. Bilateral measurements were taken from the inner skin edge to the outer margin of the abdominal muscle, and their average was used in the analysis.

### Statistical Analyses:

Data are presented as median with interquartile range or number and percent frequency, as appropriate. The comparison between groups was performed using the Chi-square or Fisher test for categorical variables, Mann–Whitney test for non-normally distributed variables and by independent T test for normally distributed variables. We used the Shapiro–Wilk test for assessing the normality of the distribution. A univariable logistic regression analysis was used to identify factors associated with the development of posttransplant diabetes. All the variables associated with the outcome (P < .05) were included into a stepwise multivariable logistic regression analysis. We considered a P value of less than 0.05 to be significant. All analyses were performed using Statistical Package for the Social Sciences (SPSS) version 29.0.

## Results

We included 478 adult kidney transplant recipients (mean age 41.2 years, range 18–71; 57.1% female) in our analysis. The details of the participant selection process are outlined in Supplementary Fig. 1. The median follow-up period was 3.65 years. Baseline demographic, laboratory and imaging outcomes of included participants are summarized at Table 1. A total of 80 patients (16.7%) have developed PTDM during the follow-up period. None of the patients had received corticosteroid-free maintenance immunosuppressive regimen. All of the patients were diagnosed with PTDM either with ongoing need for glucose lowering treatment after 120 days postoperatively or having high fasting plasma glucose and hemoglobin A1c levels > 6.5%. None of the patients were diagnosed with OGTT. Patients developing PTDM were older with higher BMI, abdominal subcutaneous adipose tissue thickness, and waist circumference and higher baseline serum glucose and triglyceride levels than patients not developing PTDM (Table 1). No differences were recorded in terms of gender, baseline comorbidity status, eGFR or serological parameters in-between groups.

**Figure 1:**
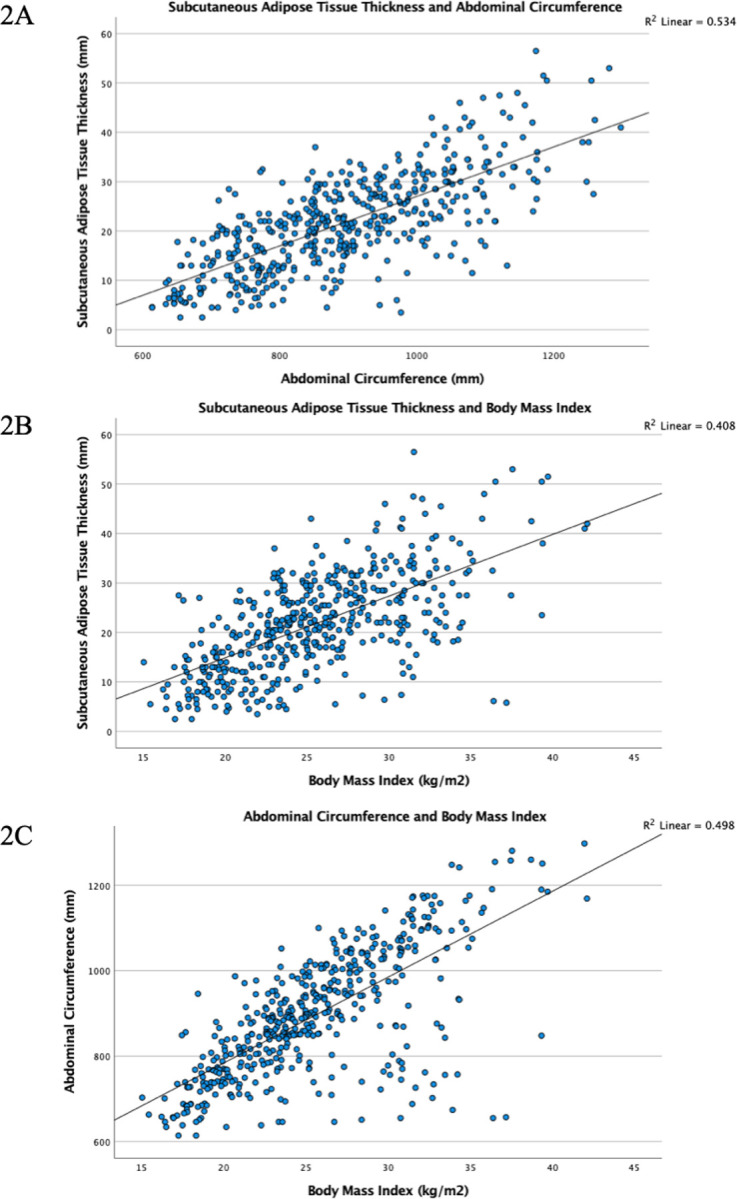
The scatterplots for the evaluation of association in-between body-mass index (BMI), abdominal waist circumference and abdominal subcutaneous adipose tissue thickness. (A) The positive correlation in-between abdominal waist circumference and abdominal subcutaneous adipose tissue thickness (R=0.53); (B) The positive correlation in-between BMI and abdominal subcutaneous adipose tissue thickness (R=0.40); (C) The positive correlation in-between BMI and abdominal waist circumference (R=0.49).

In the univariable analysis, age, BMI, abdominal subcutaneous adipose tissue thickness, abdominal waist circumference, baseline serum glucose and triglyceride levels associated with PTDM development (Table 2). After performing multivariable logistic regression analysis, age, abdominal subcutaneous adipose tissue thickness and abdominal waist circumference were statistically significant. There is also a strong positive correlation in-between those parameters were illustrated via scatterplots (Fig. 1A, 1B, 1C).

**Figure 2:**
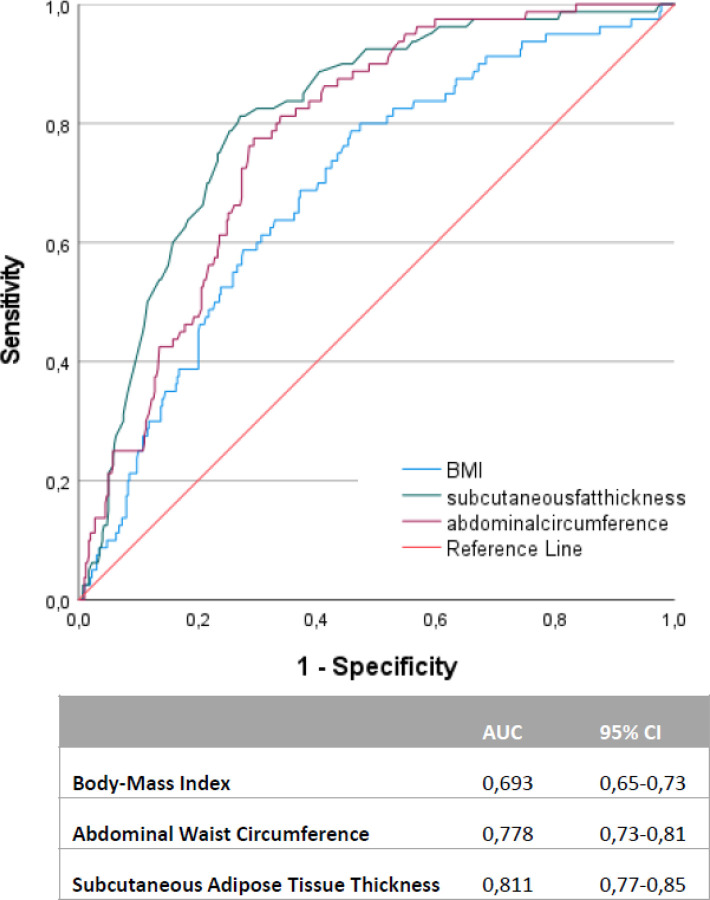
The ROC curve analysis of body mass index, abdominal subcutaneous adipose tissue thickness and abdominal waist circumference. AUC: area under the curve

The ROC curve analysis of data for BMI has suggested 24.87 kg/m^2^ as a cutoff with area under curve (AUC) of 0.693 while similar analysis for abdominal subcutaneous adipose tissue thickness has illustrated 24.75 mm as a cutoff with AUC of 0.811. Moreover, the ROC curve analysis for abdominal waist circumference illustrated 937.5 mm as a cutoff with AUC of 0.778 (Fig. 2). The pairwise comparison of the ROC curves for BMI, abdominal waist circumference and abdominal subcutaneous adipose tissue thickness has revealed the superiority of abdominal waist circumference and abdominal subcutaneous adipose tissue thickness over BMI in predicting PTDM risk (p-value < 0.001) among kidney transplant recipients (Supplementary Table 1). On the other hand, there were no statistically significant inter-difference between abdominal waist circumference and abdominal subcutaneous adipose tissue thickness (p-value = 0.17).

## Discussion

Our retrospective cohort study has demonstrated that higher pre-transplant age, BMI, abdominal waist circumference and subcutaneous adipose tissue thickness were significant predictors for PTDM among kidney transplant recipients. Furthermore, abdominal waist circumference and subcutaneous adipose tissue thickness were superior to BMI in terms of PTDM risk prediction. Even though the association of such parameters with diabetes mellitus risk in general population have recently been established [14], application of such context to transplant recipients is a novel field of research.

The prevalence of PTDM in our study population was 20.4% with a mean follow up time of 3.65 years. A large-scale retrospective study has revealed that PTDM prevalence of 7%, 10%, 13% and 21% among a total of 2.078 non-diabetic renal allograft recipients at post-transplant year 1, 3, 5 and 10, respectively [15]. Other large-scale clinical studies have revealed PTDM prevalence between 10% and 40% depending on age, ethnicity, immunosuppressive regimens, and acute rejection episodes [16, 17]. The potential risk factors for PTDM include older age, higher BMI (> 25 kg/m^2^), pre-transplant diabetes status (ie. Impaired fasting plasma glucose or impaired glucose tolerance), family history, ethnicity, certain genetic risk factors (ie. Variation in genes coding for IL-4, IL-17R, IL-7R, IL-2, KCNJ11), immunosuppressive regimens, CMV and HCV infection, deceased donor, and acute rejection episodes [18]. Kidney transplant recipients over age 45 have more than double the risk of PTDM with an even higher risk with increasing age [19] which was well correlated with our cohort. Higher pre-transplant BMI has shown to be a strong risk factor for PTDM development in multiple clinical studies. A retrospective analysis of 204 adult kidney transplant recipients has revealed that PTDM risk at months 3, 6 and 12 has increased by a factor of 1.11 (95% CI 1.0–1.23), 1.13 (95% CI: 1.03–1.24), and 1.15 (95% CI: 1.05–1.27), respectively, per unit increase in pre-transplantation BMI [20]. Our previous study involving 373 kidney transplant recipients has also illustrated a statistically significant association between higher BMI (OR = 1.13, 95% CI = 1.07–1.19 for each kg/m2 increase; p < 0.001) and age (odds ratio (OR) = 1.06; 95% confidence interval (CI) = 1.03–1.08 for each year increase; p < 0.001) and PTDM risk [7].

We have identified two novel risk factors for PTDM risk as increased abdominal waist circumference and subcutaneous adipose tissue thickness. Increased waist circumference has long been considered as a major risk factor for type 2 diabetes mellitus, though, our study is, to the best of our knowledge, the first clinical study to evaluate such an association among kidney transplant recipients. A large-scale prospective cohort study, referred as the European Prospective Investigation into Cancer and Nutrition (EPIC) study involving a total of 9.753 males and 15.491 females, has demonstrated a statistically significant association between type 2 diabetes mellitus risk and higher baseline waist circumference over a mean follow-up period of 8 years as evident from approximately 8% increase in relative risk with every 1 cm increase in waist circumference [21]. A similar pattern has been reported in multiple other clinical trials, as well [22–24]. Moreover, there are controversial data regarding the association between abdominal subcutaneous adipose tissue thickness and type 2 diabetes mellitus risk. Even though higher adiposity may hypothetically lead to hyperinsulinemia and peripheral insulin resistance through multiple mechanisms, including the release of free fatty acids entering portal circulation and inducing gluconeogenesis and hyperlipidemia, inhibition of skeletal muscle glucose uptake [25] and release of pro-inflammatory and/or pro-fibrotic cytokines [26], the clinical scenario is not that straightforward. Despite the early reports indicating higher risk for type 2 diabetes among patients with higher abdominal subcutaneous fat deposition [27–29], a large-scale clinical study involving a total of 12.137 participants in which adipose tissue accumulation was evaluated via magnetic resonance imaging has identified subcutaneous fat deposition as a protective factor for new onset diabetes in contrast to visceral fat accumulation [30]. Similarly, the protective effects of subcutaneous adipose tissue accumulation at the extremities have been validated in other studies [31, 32]. Such controversy may be attributable to lower angiogenic capacity of subcutaneous adipose tissue compared to visceral adipose tissue [33], though, there is a clear need for future clinical studies on such an issue.

Our study provides the first clinical evidence of a strong association between abdominal subcutaneous adipose tissue accumulation and PTDM risk. Moreover, our study has provided the first clinical evidence supporting the superiority of such two measures over BMI in a statistically significant manner in assessing PTDM risk. The clinical significance of waist circumference was evident especially at patients with low BMI as a few studies indicate equally greater risk for diabetes among patients of low to normal BMI (< 25 kg/m2) with a large waist circumference and overweight patients (BMI: 25–29.9 kg/m2) with a small waist circumference [21]. Such superiority may be attributable to the inability of BMI to discriminate between various different body components despite being correlated with adipose tissue percentage [34] and inability to differentiate between adipose tissue distribution or hypervolemia [35], though, evaluation of such measures are challenging and not as straightforward as BMI. Furthermore, the clinical implications of such measures over BMI in the prediction of PTDM risk are unclear despite providing more statistically significant predictive power.

Patients with higher risk for PTDM might be detected, followed, and educated regarding their risk for diabetes, even during the pretransplant evaluation. The risk categorization could be renewed during routine follow-up evaluations after the transplant, simply with waist circumference. This information might be used for patients to remind self-measurements of waist circumference at home alongside with tracking of weight. The patients with higher risk for PTDM with pretransplant tomography evaluations and developing risk with waist circumference track, might be managed with exercise, lifestyle modifications and consulted with dietitian to prevent PTDM.

The primary limitations of our study include; (a) retrospective and single-center design limiting the generalizability of our findings without any data enabling external validation of our findings; (b) lack of heterogeneity in terms of CMV and HCV serological status among transplant recipients preventing meaningful statistical analysis on serological data; (c) high percentage of patients with unknown etiology for ESKD which may or may not alter PTDM risk; (d) diagnosis of PTDM based upon fasting or random plasma glucose levels with no patients undergoing oral glucose tolerance testing which may lead to potential underestimation of true PTDM incidence; (e) lack of routine pre-transplant OGTT screening which may lead to missing the patients with pretransplant undiagnosed prediabetes and overestimation of PTDM incidence; (f) risk factors that previously established in literature, the family history of diabetes and potential effect of uricosuric agents was not included in the analysis due to retrospective design; (g) use of standardized maintenance immunosuppressive regimens preventing statistical analysis regarding the association between PTDM and calcineurin inhibitors or mTOR inhibitors without any comparison evaluating the effect of immunosuppressive medications’ trough level differences; (h) inability to perform time-to-event analysis using Cox regression due to difficulty in accurately determining the time from transplantation to the diagnosis of PTDM. Furthermore, clinical utility of our parameters, namely CT-derived abdominal waist circumference and subcutaneous adipose tissue thickness, may be limited outside of transplant centers as routine abdominal cross-sectional imaging modalities are not commonly employed for such purpose. However, the knowledge regarding substitution of CT-derived measures with anthropometric measurement is yet scarce. On the other hand, standardized evaluation of participants with abdominal computed tomography with regard to waist circumference and subcutaneous adipose tissue thickness and the inclusion of a high number of kidney transplant recipients are the major strengths of our study that enhance the quality of our research. Even though we have identified multiple significant risk factors for PTDM development, there is a clear need for future large-scale multicentered clinical trials to evaluate pre-transplant risk factors and to identify high-risk patients along with trials to evaluate therapeutic approaches such as change in lifestyle, using glucagon-like peptide-1 analogues.

## Conclusion

PTDM is associated with poor clinical outcomes, including allograft function and/or survival, major adverse cardiovascular events and all-cause mortality. We have performed a retrospective cohort study of 478 non-diabetic adult kidney transplant recipients aiming to evaluate potential determinants of PTDM risk. We found that older age and BMI, along with higher abdominal subcutaneous adipose tissue thickness and waist circumference were associated with PTDM independently. Furthermore, our study demonstrated superiority of abdominal waist circumference and abdominal subcutaneous adipose tissue thickness over BMI to predict PTDM. Large-scale prospective clinical studies are needed to evaluate additional risk factors and to identify high-risk solid organ transplant recipients.

## Supplementary Material

Supplementary Files

This is a list of supplementary files associated with this preprint. Click to download.

- SupplementaryFigure1.png
- SupplementaryTable1.docx

## Figures and Tables

**Table 1:** The baseline demographic, laboratory and imaging findings of participants.

	Total, N = 478	Post transplant DM (+), N = 80	Post transplant DM (−), N = 398	*p* value
*Demographic and Clinical Characteristics of Recipients*				
Age, year	42 (18)	49 (18)	40.5 (18)	**< 0.001**
Male, n (%)	205 (42.9)	30 (37.5)	175 (44)	0.287
Smoking, n (%)	111(23.2)	22 (27.5)	89 (22.4)	0.322
Hypertension, n (%)	361 (75.5)	67 (83.8)	294 (75.8)	0.122
Heart Failure, n (%)	27 (5.6)	3 (3.8)	24 (6.2)	0.396
**Radiologic and Anthropomorphic Features**				
BMI, kg/m2	24.98 (7.2)	28.28 (6)	24.34 (6)	**< 0.001**
Abdominal subcutaneous fat thickness, mm	21.5 (13.5)	30.25 (7.61)	19.90 (13)	**< 0.001**
Abdomen circumference, mm	886.5 (222.8)	997.50 (143.3)	858.50 (199.3)	**< 0.001**
**Liver Tests**				
AST, U/L	13 (8)	13 (10)	13 (8)	0.392
ALT, U/L	13 (8)	13 (7)	13 (9)	0.250
ALP, U/L	75 (38)	75 (54)	75 (37)	0.469
Total Bilirubin, mg/dL	0.31 (0.2)	0.30 (0.1)	0.31 (0.2)	0.156
Albumin, g/dL	4.31 (0.6)	4.30 (0.5)	4.31 (0.6)	0.857
**Complete Blood Count Parameters**				
Hemoglobin, g/dL	10.3 (2.3)	9.9 (2.2)	10.3 (2.3)	0.304
Platelets, 103/mm3	203 (85)	198 (75)	205 (87)	0.567
**Kidney Tests and Basic Metabolic Panel**				
Creatinine, mg/dL	7.1 (3)	7.0 (3.1)	7.1 (3.1)	0.911
Blood Urea Nitrogen, mg/dL	61.0 (30.3)	61.5 (36.8)	61.0 (29)	0.682
eGFR, mL/min/1.73m2	8 (4)	8 (4)	8 (4)	0.369
Baseline serum glucose, mg/dL	91 (11)	95 (12)	90 (11)	**< 0.001**
Baseline uric acid, mg/dL	6.4 (2.7)	6.2 (2.9)	6.4 (2.6)	0.872
Pre-transplant magnesium, mg/dL	2.22 (0.58)	2.21 (0.62)	2.22 (0.58)	0.945
Post-transplant magnesium, mg/dL	1.82 (0.32)	1.84 (0.35)	1.82 (0.31	0.787
TSH, mIU/L	2.1 (1.7)	1.96 (2.7)	2.11 (1.6)	0.971
**Lipid Profile**				
Triglycerides, mg/dL	138.5 (94)	157.5 (132)	135.5 (87)	**< 0.001**
Total cholesterol, mg/dL	191 (63)	195 (69)	189.5 (62)	0.821
HDL cholesterol, mg/dL	45 (20)	41.5 (17)	46 (21)	0.275
LDL cholesterol, mg/dL	121.5 (55)	121 (45)	121.5 (57)	0.416
**Infection Serology and Graft Rejection Status**				
Recipient anti CMV antibodies, n (%)	457 (95.6)	80 (100)	377 (95.2)	**0.046**
Donor anti CMV antibodies, n (%)	446 (93.3)	69 (95.8)	377 (99.2)	**0.022**
Kidney graft rejection, n (%)	42 (8.8)	8 (10)	34 (8.5)	0.675

Data are expressed as median with interquartile range or number and percent frequency, as appropriate. Bold values are statistically significant. Abbreviations: ALT, alanine aminotransferase; ALP, alkaline phosphatase; AST, aspartate aminotransferase; BMI, body mass index; CMV, cytomegalovirus; eGFR, estimated glomerular filtration rate; HDL high density lipoprotein; LDL, low density lipoprotein; TSH, thyroid-stimulating hormone.

**Abbreviations:** ALT, alanine aminotransferase; ALP, alkaline phosphatase; AST, aspartate aminotransferase; BMI, body mass index; CD, cluster of differentiation; CI, confidence interval; CMV, cytomegalovirus; CT, computed tomography; eGFR, estimated glomerular filtration rate; ESKD, end-stage kidney disease; HDL high density lipoprotein; IQR, interquartile range; LDL, low density lipoprotein; mTOR, mammalian target of rapamycin; OR, odds ratio; PTDM, post-transplant diabetes mellitus; TSH, thyroid-stimulating hormone.

**Table 2:** Univariable and multivariable predictors of post-transplant diabetes among kidney transplant recipients.

	Univariable			Multivariable		
	OR	CI %95	*p*-value	OR	CI %95	*p*-value
**Age, year**	1.058	1.036–1.081	< .001	1.052	1.025–1.052	< .001
**Body mass index, kg/m2**	1.131	1.078–1.187	< .001	0.879	0.792–0.975	0.015
**Abdominal subcutaneous fat thickness, mm**	1.117	1.084–1.151	< .001	1.112	1.064–1.162	< .001
**Abdomen circumference, mm**	1.007	1.005–1.009	< .001	1.004	1.000–1.008	0.033
**Baseline serum glucose, mg/dL**	1.040	1.017–1.063	< .001	1.023	0.997–1.051	0.088
**Triglycerides, mg/dL**	1.004	1.002–1.006	< .001	1.001	0.999–1.004	0.292

## Data Availability

Data can be shared if requested.

## Key learning points

### What was known

Post-transplant diabetes mellitus is a major comorbidity affecting 10–40% of kidney transplant recipients with significant clinical consequences including diabetic microvascular and macrovascular complications, infectious complications, allograft loss, and mortality.

### This study adds

Abdominal waist circumference and subcutaneous adipose tissue thickness, measured by abdominal CT scan, predict the development of post-transplant diabetes mellitus better than body-mass index.

### Potential impact

Baseline measurement of abdominal waist circumference and subcutaneous adipose tissue thickness may help to predict development of posttransplant diabetes mellitus. This may also facilitate early management of these patients.

Data are expressed as median with interquartile range or number and percent frequency, as appropriate. Bold values are statistically significant. Abbreviations: ALT, alanine aminotransferase; ALP, alkaline phosphatase; AST, aspartate aminotransferase; BMI, body mass index; CMV, cytomegalovirus; eGFR, estimated glomerular filtration rate; HDL high density lipoprotein; LDL, low density lipoprotein; TSH, thyroid-stimulating hormone.
